# Carboxymethyl cellulase production optimization from newly isolated thermophilic *Bacillus subtilis* K-18 for saccharification using response surface methodology

**DOI:** 10.1186/s13568-017-0331-3

**Published:** 2017-01-31

**Authors:** Muhammad Irfan, Qudsia Mushtaq, Fouzia Tabssum, Hafiz Abdullah Shakir, Javed Iqbal Qazi

**Affiliations:** 1 Department of Biotechnology, University of Sargodha, University Road, Sargodha, 40100 Pakistan; 2 Microbial Biotechnology Laboratory, Department of Zoology, University of the Punjab, New Campus, Lahore, 54590 Pakistan

**Keywords:** 16S rRNA, Cellulase, RSM, *Bacillus* sp. submerged fermentation, Saccharification

## Abstract

In this study, a novel thermophilic strain was isolated from soil and used for cellulase production in submerged fermentation using potato peel as sole carbon source. The bacterium was identified by 16S rRNA gene sequencing technology. Central composite design was applied for enhanced production using substrate concentration, inoculum size, yeast extract and pH as dependent variables. Highest enzyme titer of 3.50 ± 0.11 IU/ml was obtained at 2% substrate concentration, 2% inoculum size, 1% yeast extract, pH 5.0, incubation temperature of 50 °C for 24 h of fermentation period. The crude enzyme was characterized having optimum pH and temperature of 7.0 and 50 °C, respectively. The efficiency of enzyme was checked by enzymatic hydrolysis of acid/alkali treated pine needles which revealed that 54.389% saccharification was observed in acid treated pine needles. These results indicated that the cellulase produced by the *Bacillus subtilis* K-18 (KX881940) could be effectively used for industrial processes particularly for bioethanol production.

## Introduction

Cellulases are complex enzymes comprising of endoglucanases (EC 3.2.1.4), cellobiohydrolases (EC 3.2.1.91) and β-glucosidases (EC 3.2.1.21) which act on cellulose to produce glucose (Yi et al. 1999; Bhat and Bhat 1997). Cellulase production has been observed from many aerobic bacterial strains like *Bacillus megaterium* (Shahid et al. 2016), *B. subtilis* (Heck et al. 2002), *B. cereus* (Yopi et al. 2016), *B. circulans* (Kim 1995), *Cellulomonas fimi*, *Cellulomonas flavigena* (Sami and Akhtar 1993), *Cellulomonas uda* (Nakamura and Kitamura 1983), *Pseudomonas fluorescens* and some anaerobic bacteria like *Bacteroides cellulosolvens*, *Clostridium thermocellum*, *Fibrobacter succinogenes*, and *Ruminucoccus albus* (Lopez-Contreras et al. 2004; Shen et al. 1996).

Various techniques have been employed for production of cellulase enzyme from fermentation systems. Most commonly used are submerged and solid state fermentations which differ from each other with respect to environmental conditions particularly level of free water present in the medium (Mazutti et al. 2010; Pandey 2003). Optimization of process parameters is necessary to enhance the enzyme production in fermentation system. Two approaches are used to optimize these parameters which are one factor at a time (OFAT) and response surface methodologies (RSM). The first approach is time consuming and further is not considered as accurate whereas the second technique is widely used due to its advantages (Li et al. 2006; Jeya et al. 2010).

Different substrates are used for production of enzymes from fermentation processes. Most frequently employed substrates are agricultural wastes due to their abundant availability. Most commonly used agroindustrial wastes are wheat bran, sugarcane bagasse, rice straw, wheat straw, corn cobs, soy bran, rice husk, coffee husk and barley (Sanchéz 2009). The enzymes particularly cellulases produced from these substrates by fermentation technology are widely employed in various industrial processes such as in textile, pulp and paper, detergent and food industries (Graminha et al. 2008; Hebeish et al. 2009). This main objective of this study was (1) isolation and identification of potential cellulase producer bacterial strain (2) utilization of potato peel as substrate optimize process parameters by RSM and (3) application of cellulase for saccharification of pine needles to produce sugars.

## Materials and methods

### Isolation and Molecular identification of bacterium

The bacterium was isolated using standard procedures, and purified by repeatedly streaking the well isolated colonies on nutrient agar and then the growth stored at 4 °C on the agar slant. The detailed procedure of molecular identification of the bacteria has been described in an earlier report (Chaudhary et al. 2009). The sequence obtained was aligned using CLUSTAL W 1.81 (Thompson et al. 1994). The Phylogenetic tree was constructed by Neighbor-Joining method using MEGA 5.0 (Molecular Evolutionary Genetics Analysis, version 5.0) software (Tamura et al. 2011).

### Enzyme production

Self-designed fermentation medium with 1 g potato peel powder was taken in 250 ml Erlenmeyer flask capacity and autoclaved at 121 °C, for 15 min at 15 Psi pressure. After sterilization, the flasks were allowed to cool at room temperature and 1 ml of the vegetative cell culture was transferred aseptically to each of the fermentation flasks. After inoculation, the flasks were incubated at 50 °C with agitation speed of 120 rpm for 24 h of fermentation period. After the termination of the fermentation period, the fermented broth was filtered through muslin cloth followed by centrifugation (Sigma 2–16 PK) for 10 min at 10,000×*g* and 4 °C for the removal of cell mass and unwanted particles. The clear cell free extract obtained after centrifugation was used as a crude source of enzyme. Triplicate readings were taken for each of the experiment.

### Carboxymethyl cellulase assay

Carboxymethyl cellulase activity was measured as described by Ghosh (1987). Reaction mixture containing 0.5 ml of 1% CMC (prepared in 0.05 M citrate buffer pH 5) and 0.5 ml of the crude enzyme solution was incubated at 50 °C for 30 min. After incubation, 1.5 ml of DNS solution was added to stop the reaction and test tube was boiled for 10 min in a water bath. Absorbance was taken at 540 nm using spectrophotometer (Spectrophotometer Cecil, CE 2042). One unit (U) of enzyme activity was defined as the quantity of enzyme, which released 1 µmol of glucose under the standard assay conditions.

### Saccharification of Pine needles

In 500 ml flask twenty-five milliliter of culture filtrate having carboxymethyl cellulase activity of 3.77 ± 0.11 IU/ml with 1% pretreated pine needles (1% H_2_SO_4_/NaOH) was incubated in a shaking water bath at 50 °C with agitation speed of 140 rpm for 8 h. After termination of enzymatic hydrolysis the material was centrifuged at 10,000 rpm for 10 min. The supernatant was removed for sugar content analysis. Saccharification (%) was calculated using the following formulae (Irfan et al. 2016).

$${\text{Saccharification }}\left( \% \right) \, = \frac{{Reducing\,sugars\,released\left( {\rm mg\,ml} \right)}}{{Substrate\,used\left( {\rm mg\,ml} \right)}} \times 100$$

### Experimental design

In order to optimize process conditions for cellulase production, central composite design (CCD) was used. The independent variables used were substrate concentration (X_1_), inoculum size (X_2_) yeast extract (X_3_) and pH (X_4_) and their levels are mentioned in Table 1. This design is most suitable for quadratic response surface and generates second order polynomial regression model. The relation between actual and coded values was described by the following equation

1 $$x_{i} = \frac{{X_{i} - X_{ \circ } }}{{\Delta X_{i} }}$$

**Table 1:** Levels and codes of variables used for CCD

Parameter	Code	Levels				
		−2	−1	0	+1	+2
Substrate conc. (%)	X_1_	0.5	1.0	1.5	2.0	2.5
Inoculum size (%)	X_2_	1	2	3	4	5
Yeast extract (%)	X_3_	0.2	0.6	0.8	1.0	1.2
pH	X_4_	4.5	5	5.5	6	6.5

where *x*_*i*_ and *X*_*i*_ are the coded and actual values of an independent variable, *X*_*o*_ is the actual value of the independent variable at the center point and Δ*X*_*i*_ is the magnitude of change of *X*_*i*_The response was calculated from the following equation using STATISTICA software (99th edition).

2 $${\text{y }} = \beta_{ \circ } + \mathop {\mathop \varSigma \limits^{k} }\limits_{i = 1} + \mathop {\mathop \varSigma \limits^{k} }\limits_{i = 1} \beta_{i} X_{i}^{2} + \mathop \varSigma \limits_{i} \mathop \varSigma \limits_{j} \beta_{1j} X_{i} X_{j}$$

where Y is the response, k is the number of variables, *β*_0_ is the intercept, X_i_ and X_j_ are independent variables, *β*_i_, is the *i*th linear coefficient, *β*_ii_ is the *i*th quadratic coefficient and *β*_ij_ is the interaction coefficient.

### Effect of pH on CMCase activity

The optimum pH of the crude CMCase was determined by incubating crude enzyme with substrate (1%CMC) prepared in appropriate buffers; 0.05 M citrate buffer (pH 3.0 to 6.0), 0.05 M sodium phosphate buffer (pH 6.0 to 8.0), 0.05 M Tris–HCl (pH 8.0 to 9.0) and 0.05 M glycine-NaOH (pH 9.0 to 11.0). Crude enzyme mixture in these pH buffers were incubated for 30 min at 50 °C. By using DNS method, CMCase activity was assayed.

### Effect of temperature on CMCase activity

The effect of temperature on CMCase activity was determined by incubating crude enzyme mixture in 1% CMC-Na in 0.05 M sodium phosphate buffer (pH 7) at temperature ranging from 30 to 100 °C. After incubation, the enzyme activity was checked by standard assay as described earlier.

### Statistical analysis

The data obtained after experimentation was statistically evaluated using ANOVA at significance level of p < 0.05 by using computer based program SPSS.

## Results

**Fig. 1:**
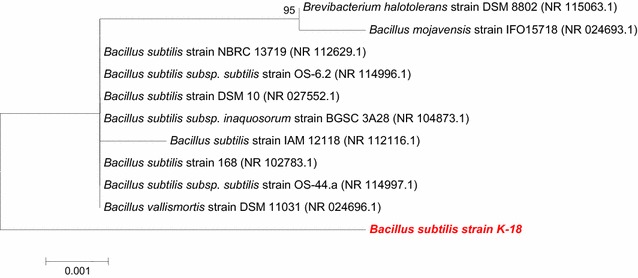
Phylogenetic analysis of newly isolated *Bacillus subtilis* K-18 using neighbor-joining method

In this study a novel cellulolytic bacterium *Bacillus subtilis* K-18 was isolated from soil. The bacterium was identified by 16S rRNA gene sequencing technology and the sequence obtained was submitted in gene bank under accession number of KX881940 possessing high homology (99%) with different strains of *Bacillus subtilis* (Fig. 1). Response surface methodology was used to optimize process variables for cellulase production in submerged fermentation using potato peel as sole carbon source. Four variables i.e. substrate concentration (X_1_), inoculum size (X_2_), yeast extract concentration (X_3_) and pH (X_4_) with five different levels (Table 1) were optimized by central composite design for cellulase production. Optimization results (Table 2) reveals that maximum enzyme production of 3.50 ± 0.11 IU/ml was achieved with 2% substrate concentration, 2% inoculum size, 1% yeast extract, pH 5.0 and incubation temperature of 50 °C for 24 h of fermentation period. The predicted enzyme yield under these conditions was 3.13 IU/ml which was little less than observed value. The enzyme activity was calculated using polynomial regression equation (Eq. 3) where Y is the yield of cellulase activity (IU) whereas X_1_, X_2_, X_3_ and X_4_ represent substrate concentration, inoculum size, yeast extract and pH, respectively.

3 $$\begin{aligned} {\text{Y }}\left( {{\text{CMCase activity}},{\text{ IU}}} \right) &= \, - 9. 7 3 3 9 3 { } + { 4}. 4 6 1 1 2 {\text{ X}}_{ 1} + 0. 2 6 3 1 7 {\text{ X}}_{ 2} + { 5}. 9 4 5 7 5 {\text{ X}}_{ 3} \\ & \quad + { 1}. 4 5 4 60{\text{ X}}_{ 4} - 0.3 9 9 8 2 {\text{ X}}_{ 1}^{ 2} + 0.0 4 4 3 2 {\text{ X}}_{ 2}^{ 2} + 0. 70 6 9 3 {\text{ X}}_{ 3}^{ 2} - 0.0 3 1 6 4 {\text{ X}}_{ 4}^{ 2} \\ & \quad + 0.0 3 6 4 9 {\text{ X}}_{ 1} *{\text{X}}_{ 2} + 0.0 3 20 9 {\text{ X}}_{ 1} *{\text{X}}_{ 3} - 0. 5 9 6 7 6 {\text{ X}}_{ 2} *{\text{X}}_{ 3} - 0.30 9 2 2 {\text{ X}}_{ 1} *{\text{X}}_{ 4} \\ & \quad - 0.0 2 6 3 5 {\text{ X}}_{ 2} *{\text{X}}_{ 4} - 0. 7 2 800{\text{ X}}_{ 3}\, *{\text{X}}_{ 4} \end{aligned}$$

**Table 2:** Effect of different variables on cellulase production through CCD

Run#	Substrate conc. (X_1_)	Inoculum size (X_2_)	Yeast extract % (X_3_)	pH (X_4_)	Enzyme activity (IU/ml)		Residual value
					Observed	Predicted	
1	0.5	3	0.8	5.5	0.32	0.39	−0.074
2	1.5	3	0.8	6.5	1.60	1.61	−0.012
3	1.0	4	1.0	5.0	1.10	1.19	−0.09
4	1.5	3	0.2	5.5	2.01	2.09	−0.08
5	1.5	3	0.8	5.5	1.57	1.88	−0.31
6	2.5	3	0.8	5.5	2.57	2.83	−0.26
7	1.0	2	1.0	6.0	1.40	1.45	−0.05
8	1.5	3	1.2	5.5	1.96	2.11	−0.15
9	1.5	1	0.8	5.5	2.10	2.30	−0.20
10	1.0	2	1.0	5.0	1.60	1.65	−0.05
11	1.0	2	0.6	5.0	1.30	1.05	0.25
12	2.0	4	0.6	6.0	2.70	2.41	0.28
13	1.0	4	0.6	6.0	1.20	1.45	−0.25
14	2.0	4	1.0	6.0	1.84	1.97	−0.13
15	2.0	2	1.0	6.0	2.50	2.36	0.13
16	1.5	3	0.8	4.5	1.70	2.02	−0.32
17	2.0	4	0.6	5.0	2.80	2.63	0.16
18	1.0	4	1.0	6.0	1.20	0.93	0.26
19	1.0	2	0.6	6.0	1.60	1.46	0.13
20	1.0	4	1.0	5.0	1.40	1.19	0.20
21	2.0	4	1.0	5.0	2.89	2.80	0.09
22	1.5	5	0.8	5.5	1.83	1.96	−0.13
23	2.0	2	0.6	5.0	2.41	2.44	−0.03
24	1.5	3	0.8	5.5	2.10	1.88	0.21
25	1.0	4	0.6	5.0	1.20	1.10	0.09
26	2.0	2	1.0	5.0	3.50	3.13	0.36

The results were analyzed by ANOVA and shown in Table 3. The model used in this study was significant having Fisher’s test value of 8.781174. In this study some parameters were found to be significant, whereas others were not significant for cellulase production in submerged fermentation. The coefficient of determination for cellulase activity was calculated as 0.958056 which can explain 95.8% variation in response and only 4.2% variation was not explained by the model. The R^2^ and adjusted R^2^ values were 0.917871 and 0.813344, respectively.

**Table 3:** Analysis of variance of response surface quadratic model for cellulase production

Effect	SS	df	MS	F	P
Model	11.02376	14	0.787412	8.781174	0.000460
X_1_	0.495223	1	0.495223	5.522699	0.038484
X_1_^2^	0.079875	1	0.079875	0.890763	0.365539
X_2_	0.000630	1	0.000630	0.007027	0.934699
X_2_^2^	0.066383	1	0.066383	0.740296	0.407928
X_3_	0.227668	1	0.227668	2.538943	0.139376
X_3_^2^	0.061811	1	0.061811	0.689311	0.424052
X_4_	0.106695	1	0.106695	1.189860	0.298675
X_4_^2^	0.004848	1	0.004848	0.054070	0.820397
X_1_*X_2_	0.016194	1	0.016194	0.180592	0.679059
X_1_*X_3_	0.006181	1	0.006181	0.068927	0.797758
X_2_*X_3_	0.251767	1	0.251767	2.807690	0.121973
X_1_*X_4_	0.302941	1	0.302941	3.378387	0.093191
X_2_*X_4_	0.003314	1	0.003314	0.036958	0.851053
X_3_*X_4_	0.351152	1	0.351152	3.916025	0.073408
Error	0.986375	11	0.089670		

**Fig. 2:**
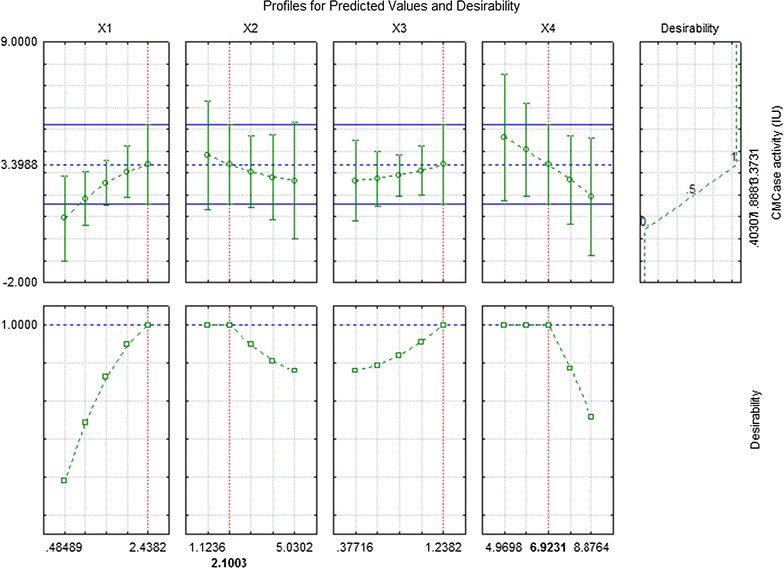
Desirability chart for CMCase production by *Bacillus subtilis* K-18 in submerged fermentation using response surface methodology

**Fig. 3:**
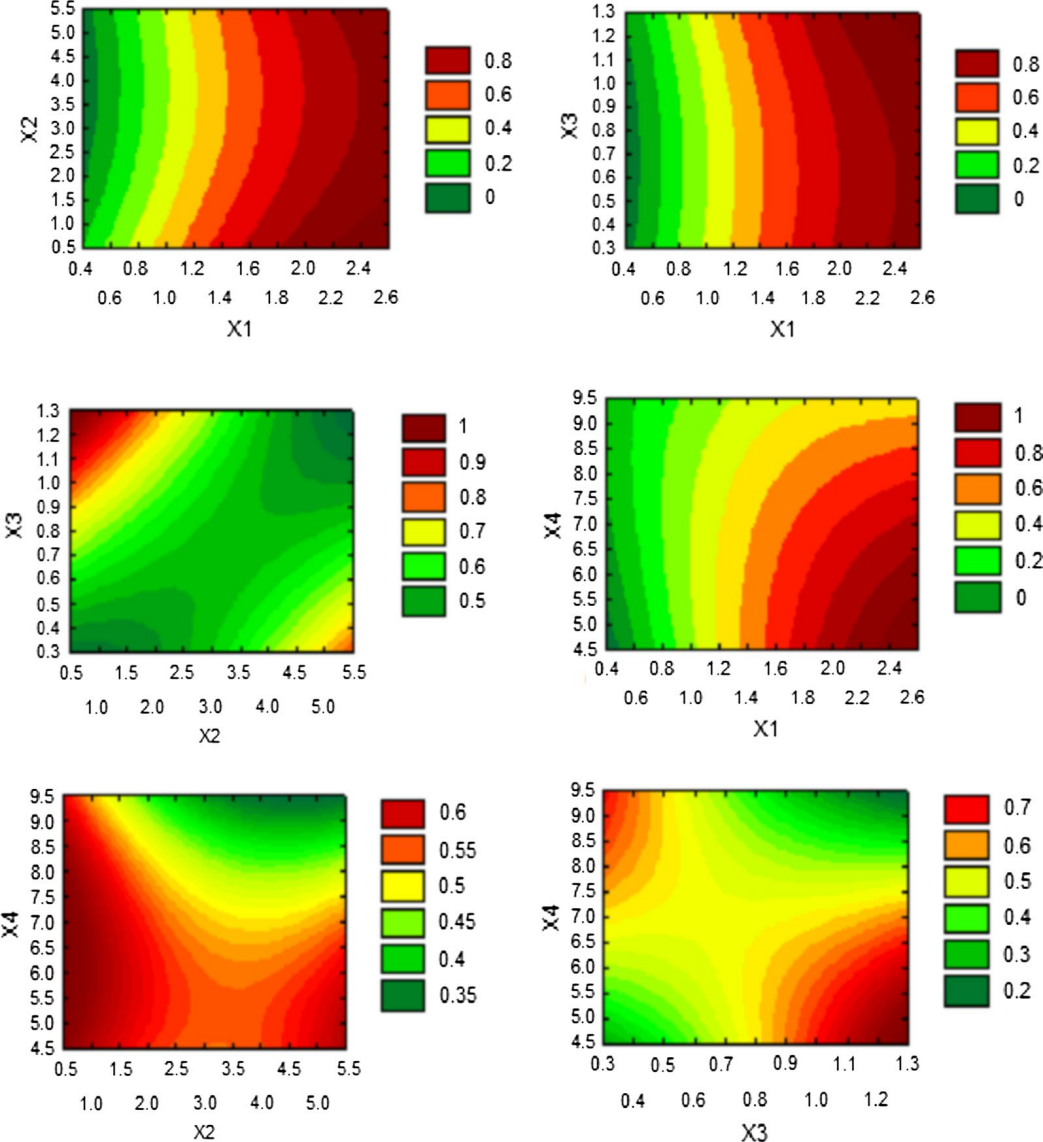
Contour plot of different variables for CMCase production from newly isolated *B. subtilis* K-18 (*X1* substrate conc.,* X2* inoculum size,* X3* yeast extract,* X4* pH)

Figure 2 represents the desirability chart for cellulase production in submerged fermentation using central composite design of response surface methodology. This chart showed that substrate concentration of 1.4615%, inoculum size of 3.0769%, yeast extract 0.80769% and pH of 6.9231 could yield cellulase activity up to 3.37 IU which was further confirmed by repeated experimentation. It is important to note that different cellulolytic bacterial species/strains yield varying titer of cellulases. The interaction effect of substrate concentration, inoculum size, yeast extract and pH is illustrated in contour and surface plots as shown in Fig. 3. These results showed that all the parameters with their interactions have critical effect on cellulase production in submerge fermentation. Substrate concentration had significant effect on cellulase production by *B. subtilis* in submerged fermentation.

**Fig. 4:**
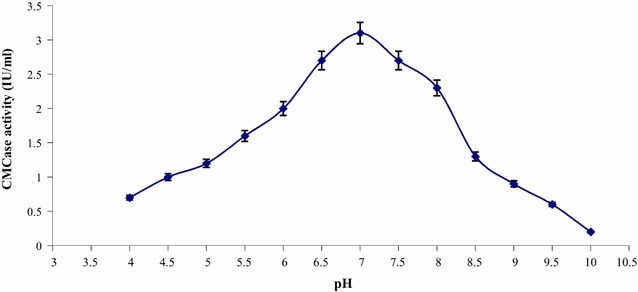
Effect of pH on CMCase activity of *B.subtilis* K-18

**Fig. 5:**
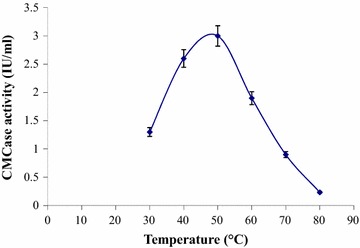
Effect of temperature on CMCase activity of *B.subtilis* K-18

Effect of pH and temperature was studied on crude CMCase activity produced from *B. subtilis* K-18 in submerged fermentation. Results (Fig. 4) revealed that the crude CMCase exhibited optimum pH of 7.0. The CMCase activity was decreased as the pH increased towards alkalinity. Further increased in pH or acidic pH lowered CMCase activity. When temperature profile of the crude CMCase was studied, it was found that (Fig. 5) incubation temperature of 50 °C favored maximum CMCase activity revealing its thermophilic nature. Increment in temperature up to 100 °C leads decline in enzyme activity.

**Fig. 6:**
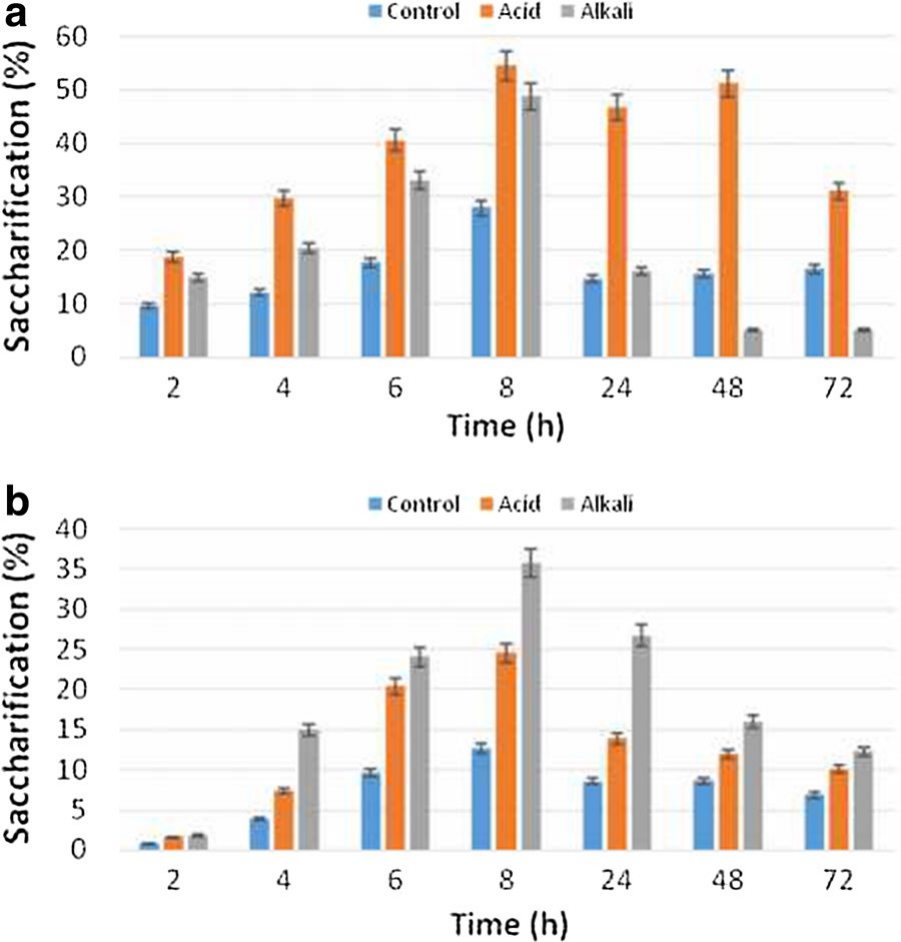
Saccharification of pine needles by **a** commercial enzyme and **b** indigenously produced cellulase enzyme

**Fig. 7:**
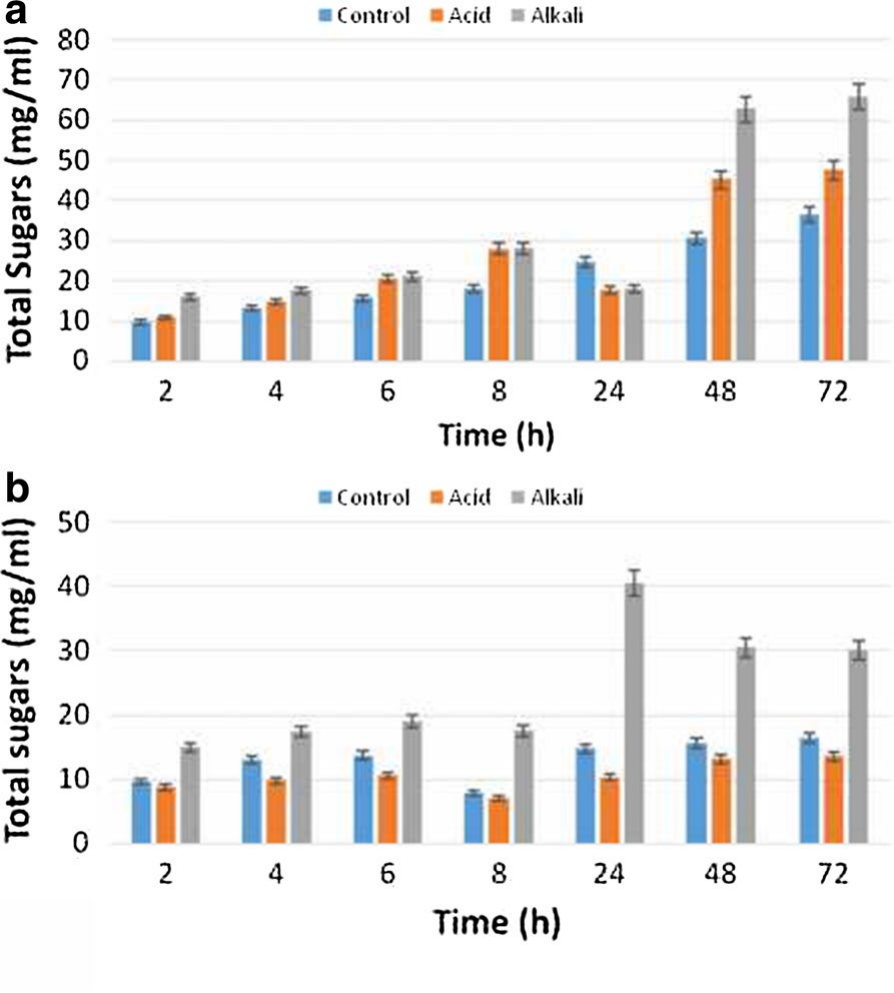
Total sugars produced from Pine needles by **a** commercial enzyme **b** indigenously produced enzyme

The cellulase enzyme produced by the *Bacillus subtilis* K-18 (KX881940) was tested for saccharification of pinus needles for production of fermentable sugars. Three different categories (control, H_2_SO_4_ and NaOH) of treated pine needles were employed for saccharification by commercial enzyme and indigenously produced cellulase enzymes. The results (Fig. 6a) revealed that maximum saccharification (54.38%) was obtained in H_2_SO_4_ treated pine needles as compared to NaOH and untreated samples using commercial cellulase enzyme whereas indigenously produced cellulase enzyme yield 35.7% saccharification (Fig. 6b) of NaOH treated pine needles which was higher as compared to acid treated and untreated samples. The saccharification process was observed under different time interval and it was found that 8 h of incubation at 50 °C yielded maximum saccharification. Level of total sugars production in saccharification process increased with increase in incubation time. 72 h of incubation time yielded highest (65.73 mg/ml) amount of total sugars with commercial enzyme using 3% NaOH treated pine needles (Fig. 7a). Indigenously produced cellulase enzyme yielded 40.48 mg/ml of total sugars from 3% NaOH treated pine needles after 24 h of incubation time at 50 °C (Fig. 7b).

## Discussion

This study dealt with cellulase production from locally isolated thermophilic strain of *Bacillus subtilis* K-18 (KX881940) in submerged fermentation. Potato peels as a waste was used as sole carbon source and production was optimized through central composite design of response surface methodology. In this context we got the maximum production of cellulase under optimized conditions of 2% substrate concentration, 2% inoculum size, 1% yeast extract, pH 5.0 and incubation temperature of 50 °C for 24 h of fermentation period. For example previous studies reported that maximum CMCase production was achieved at initial medium pH of 7.0 and inoculum size of 2% from locally isolated cellulolytic strain (Safdar et al. 2013). Vasudeo and Lew (2011) obtained maximum yield of cellulase from B. *amyloliquefaciens* UNPDV-22 at pH of 5.25, and inoculum size of 4.95% (v/v) optimized through central composite design of response surface methodology. Initial medium pH of 8.0 and inoculum size of 3% has been reported for maximum cellulase production by *Bacillus subtilis* in submerged fermentation (Gautam and Sharma 2014). A strain of *Bacillus subtilis* BY-2 isolated from the pig intestine exhibited maximum cellulase production at initial medium pH of 5.5 and inoculum size of 4% in submerged fermentation (Yang et al. 2014).

Significant influence of different process parameters for cellulolytic enzyme production in solid state fermentation has also been reported in the previous study wherein potato peels were employed as substrate and various parameters were optimized by response surface methodology (dos Santos et al. 2012). Some bacteria like *Cellulomonas* sp. possess considerable potential for utilizing potato waste as substrate for cellulase production in submerged fermentation (Irfan et al. 2012). Likewise some fungi also exhibit potential for utilizing potato peel residues as a substrate for cellulase production (Taher et al. 2016).

In this study, the optimum pH and temperature of crude CMCase enzyme was found 7.0 and 50 °C produced from *B. subtilis* K-18 under submerged fermentation. The CMCase produced from this strain was found to be active at neutral pH and thermophilic. Rawat and Tewari (2012) reported cellulase from *Bacillus subtilis* strain LFS3 having optimum pH and temperature of 4.0 and 60 °C respectively. Another study also revealed that cellulase produced from *Bacillus* sp. having optimum pH and temperature of 6 and 50 °C (Vijayaraghavan and Vincent 2012). Shu-Bin et al. (2012) stated that *Bacillus subtilis* pa5 produced cellulase enzyme having optimum pH and temperature of 7 and 50 °C respectively.

The results revealed the total sugars and saccharification yield was higher in treated substrates as compared to control (untreated pine needles) which depicted that pretreatment effectively degraded the lignin component and exposed maximum cellulose for subsequent enzyme attack. Similar findings have also been reported earlier stating that pretreated samples yield more degradation as compared to untreated substrates (Sharma et al. 2011). Tandon et al. (2012) reported only 12.81% hydrolysis rate of NaOH + H_2_O_2_ treated pine needles with indigenously produced cellulase and xylanase from *P. notatum*-102 obviously; this yield is much less than results of our study. Further cellulolytic potential of the bacterium *Bacillus subtilis* K-18 (KX881940) in the potato peel substrate, which mainly comprised of starch is suggestive to verify the enzyme yield while employing cellulosic substrates. Such attempts will likely lead to enhanced enzyme titer.

## Abbreviations

CMC: carboxymethyl cellulose
RSM: response surface methodology
